# Cholecystoduodenal Stenting: An Option in Complicated Acute Calculous Cholecystitis in the Elderly Comorbid Patient

**DOI:** 10.1155/2018/1609601

**Published:** 2018-01-21

**Authors:** Brady Chapman Bonner, Nicholas I. Brown, Varghese Pynadath Joseph, Manju Dashini Chandrasegaram

**Affiliations:** ^1^Department of General Surgery, The Prince Charles Hospital, Brisbane, QLD, Australia; ^2^School of Medicine, The University of Queensland, Brisbane, QLD, Australia; ^3^Department of Radiology, The Prince Charles Hospital, Brisbane, QLD, Australia; ^4^Wesley Medical Imaging, Auchenflower, QLD 4006, Australia

## Abstract

We describe the course of an 84-year-old lady with acute calculous cholecystitis. She was unable to have a cholecystectomy due to multiple comorbidities including morbid obesity, type 2 diabetes, Guillain–Barrè syndrome, chronic sacral pressure ulcer, and severe cardiac disease. Conservative treatment with intravenous antibiotics was initially successful; however, she subsequently re-presented with an empyema of the gallbladder. She was readmitted for further intravenous antibiotics and underwent percutaneous gallbladder drainage. The patient did not want a permanent catheter for drainage, nor the prospect of repeat drainage procedures in the future for recurrent cholecystitis. Following a discussion of the rationale and risks involved with other minimally invasive techniques, she underwent cholecystoduodenal stent placement following disimpaction and removal of cystic duct stones. The procedure restored antegrade gallbladder drainage, and at 18 months she remains symptom-free from her gallbladder. Long-term management of recurrent cholecystitis in elderly comorbid patients commonly includes permanent cholecystostomy or repeated percutaneous gallbladder drainage, both of which can be poorly tolerated. Permanent cholecystoduodenal stenting is a reasonable alternative in carefully considered patients in whom the benefits outweigh the risks. We describe our experience with cholecystoduodenal stenting and discuss some of the concerns and considerations with this technique.

## 1. Introduction

The management of acute cholecystitis in elderly or infirm patients unfit for surgery is challenging. Some patients with good quality of life remain high-risk surgical candidates. While a percutaneous cholecystostomy can be an appropriate temporizing measure for patients with significant comorbidities, it does not address the longer-term problem of recurrent cholecystitis. Although a permanent cholecystostomy catheter may be sufficient acute treatment in severely debilitated patients, others may not tolerate permanent drain placement or repeat percutaneous interventions for recurrent cholecystitis.

We describe percutaneous cholecystoduodenal stenting following cystic duct stone disimpaction and removal. The procedure is aimed at preserving the patency of the cystic duct to prevent recurrent obstructive cholecystitis. We discuss the potential problems with this approach and explore its use in a selected group of patients.

## 2. Case Presentation

An 84-year-old lady presented to our emergency department with fevers, rigors, and right sided abdominal pain on a background of morbid obesity, type 2 diabetes, Guillain–Barrè syndrome, a chronic sacral pressure ulcer, ischaemic heart disease, congestive cardiac failure, a previous cardiac arrest, mitral regurgitation, atrial fibrillation, visual impairment, chronic lower limb oedema, and osteoarthritis. She was on numerous medications including rivaroxaban, metformin, insulin, digoxin, metoprolol, and pregabalin. She had significant mobility issues due to her Guillain–Barrè syndrome and mobilized with a wheeled walker.

On examination, she was febrile and tachycardic with tenderness in the right upper quadrant and localised guarding. White cell count was elevated (33.5 × 10^9^/L, reference range 3.5–11 × 10^9^/L), as was the bilirubin (45 *μ*mol/L, reference range < 20 *μ*mol/L), conjugated bilirubin (8 *μ*mol/L, reference range < 4 *μ*mol/L), and gamma-GT (42 U/L, reference range < 38). She underwent a computed tomography (CT) scan and an ultrasound, both of which showed a grossly distended and thickened gallbladder containing numerous gallstones, with a distended bile duct and inflammatory changes extending to the ampulla of Vater.

She was admitted with severe acute cholecystitis and was managed with gut rest and intravenous antibiotics. The patient's medical background of congestive cardiac failure, ischaemic heart disease, atrial fibrillation, mitral regurgitation, and a previous cardiac arrest rendered her a high-risk surgical candidate. This in addition to her morbid obesity, diabetes, chronic large sacral ulcer, severe lower limb oedema, and poor mobility made her a poor operative candidate. It was therefore decided to pursue conservative management. Clinical improvement occurred at the time that she was managed nonoperatively in keeping with her wishes to avoid any procedures that could lead to her functional decline. She improved during the course of the next 6 days, and she was discharged on oral antibiotics with a normal white cell count and pain free.

She was reviewed in the outpatient clinic approximately 4 weeks following her admission. Although she had been feeling well and remained pain free, examination revealed a tender, firm palpable mass in the right upper quadrant. The patient declined further investigations at this stage but agreed to return for further imaging and tests the following week.

**Figure 1:**
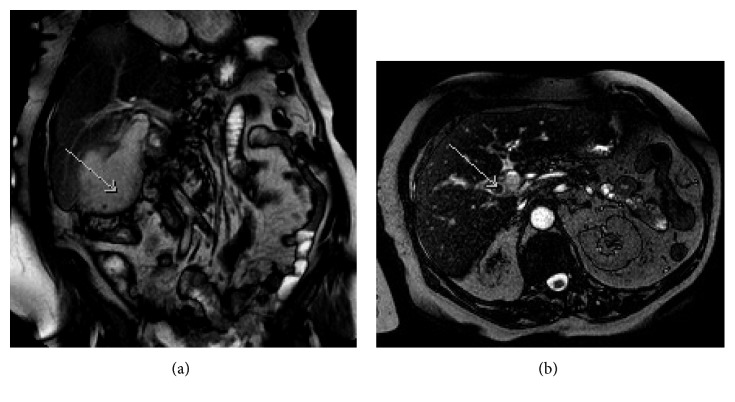
Coronal MRI image demonstrating large gallbladder empyema (a), and transverse MRI image section demonstrating 12 mm obstructing gallstone in the cystic duct (b).

During the following week, her condition deteriorated, and she was immediately admitted. Her white cell count was elevated, and a magnetic resonance cholangiopancreatography (MRCP) revealed an empyema of the gallbladder (Figure 1). The gallbladder was grossly distended and contained multiple stones. There was an obstructing calculus in the cystic duct (Figure 1); however, the biliary tree was nondilated. She was commenced on intravenous antibiotics, and an 8-French Navarre (Bard, Arizona, USA) catheter cholecystostomy was inserted percutaneously using ultrasound guidance, which immediately drained 250 mL of purulent, bilious fluid.

On day seven of her admission, a fluoroscopic cholecystogram revealed multiple gallstones with a 12 mm obstructing calculus in the cystic duct. Unfortunately the catheter became dislodged during this procedure, and another percutaneous catheter was inserted. An 8-French Britetip sheath (Cordis, Baar, Switzerland) was inserted into the gallbladder, and a catheter and guide wire were then used to disimpact the stones. A Fogarty balloon (Edwards Lifesciences, California, USA) cleared other small stones from the cystic duct. Numerous stones were then fragmented with a stone retrieval basket and removed from the gallbladder; however, the larger disimpacted calculi could not be retrieved. A pigtail drain was left in situ, with only a small contained leak noted from the initial cholecystostomy site.

The patient continued to experience pain and low-grade fevers for several days. The 24 hourly drain outputs in the five days following the second drain insertion continued to decrease and were 500 mL, 250 mL, 20 mL, 100 mL, and 40 mL, respectively.

While an interval laparoscopic cholecystectomy was considered, the patient remained a high-risk surgical candidate. The patient was also keen to pursue less invasive options as she did not want anything that could further compromise her baseline function.

**Figure 2:**
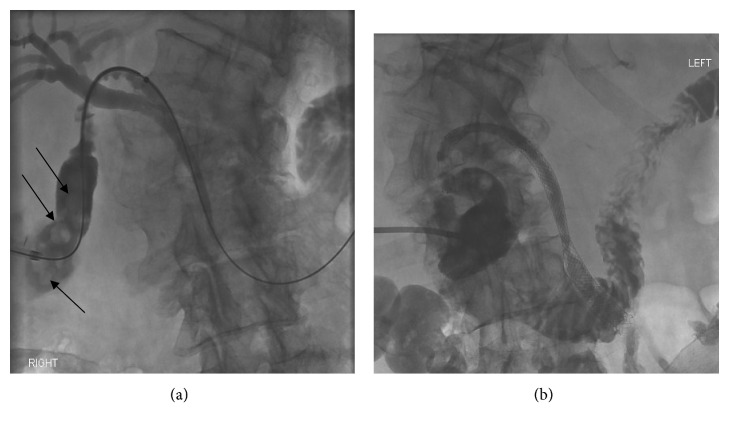
Fluoroscopy-guided insertion of a collapsed 6 mm × 60 mm bare metal stent into the cystic duct prior to deployment (a). Note the round filling defects, consistent with gallbladder calculi (arrows). Subsequent successful deployment of a 6 mm × 60 mm bare metal stent in the cystic duct, and an 8 mm × 100 mm bare metal stent in the common bile duct and duodenum, with antegrade contrast clearance (b).

After a lengthy discussion, the patient declined insertion of a permanent percutaneous drainage catheter as a definitive solution, but sought alternative strategies to avoid repeat drainage procedures for future episodes cholecystitis. We discussed the rationale and the risks involved with cholecystoduodenal stenting, which was performed on day 13 of her admission. A cholecystogram was performed to guide insertion of a catheter and wire to cannulate the cystic duct under fluoroscopic guidance. A balloon was once again used to remove obstructing stones. A 6 mm × 60 mm bare metal stent was inserted into the cystic duct (Figure 2), and an 8 mm × 100 mm bare metal stent was inserted from the mid-cystic duct through the ampulla of Vater into the duodenum. Rapid antegrade clearance of contrast from the gallbladder was then demonstrated on cholecystogram (Figure 2). An 8-French cholecystostomy drain was capped and left in situ. A repeat cholecystogram the next day demonstrated that the stents remained patent with prompt antegrade drainage of contrast, and the cholecystostomy drain was removed.

**Figure 3:**
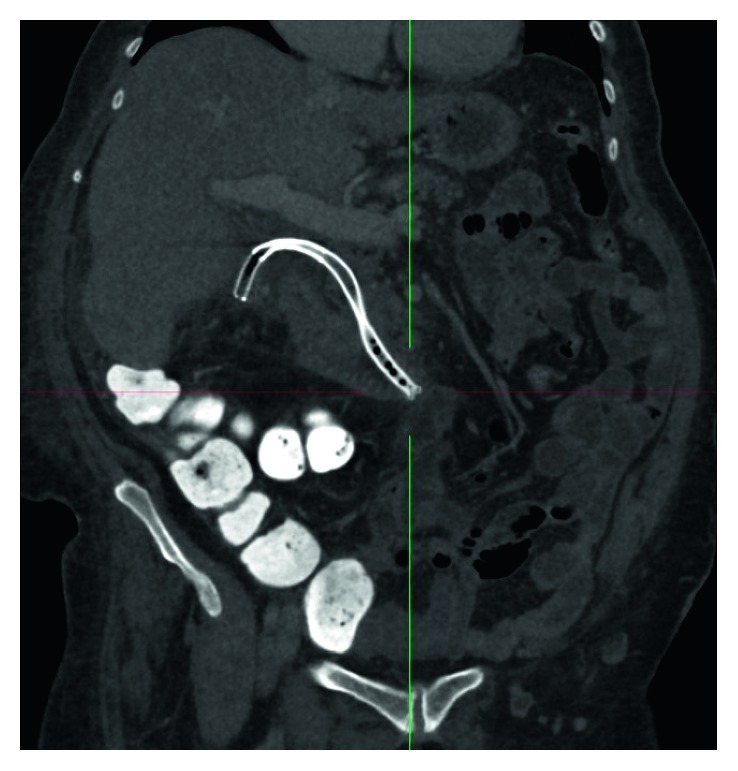
CT scan two days after stent insertion demonstrating appropriate positioning and relative size of the bare metal stents in the cystic and common bile ducts.

The patient experienced ongoing fevers for 48 hours, likely due to haematogenous septic showering from the stent insertion procedure. The brief septic episode may also have been from minor leakage from the cholecystostomy. A CT scan confirmed appropriate positioning and sizing of the metallic stents (Figure 3) and revealed a collapsed gallbladder with no discrete collection. The patient became afebrile and her pain resolved, along with normalization of her inflammatory markers and white cell count.

During her admission, the patient required ongoing management of her large sacral pressure area and skin breakdown from bilateral leg swelling which had been a preexisting chronic problem. She was discharged on day 22 from admission. She was followed up at two weeks, three months, six months, 11 months, and 18 months after discharge and remained asymptomatic. Poor mobility, bilateral limb swelling, and progression of her Guillain–Barrè syndrome remain ongoing clinical challenges for the patient.

## 3. Discussion

Laparoscopic cholecystectomy is the standard of care for acute calculous cholecystitis [1, 2]; however, it is associated with higher morbidity and mortality in elderly and comorbid patients [3]. The 2007 and updated 2013 Tokyo Guidelines (TG13) [4–13] are a widely accepted standard for systematically diagnosing, assessing, and managing acute calculous cholecystitis (ACC) and acute cholangitis based on severity scoring systems [1]. Current guidelines recommend intravenous antibiotics and percutaneous gallbladder drainage/cholecystostomy (PTGBD) in high-risk patients and patients who are inoperable [1, 11–13]. This is appropriate in moderate to severe cholecystitis when conservative treatment fails. PTGBD is a safe, effective, and appropriate treatment for elderly patients with acute cholecystitis who are poor borderline surgical candidates, with an associated procedural mortality rate of 0.36% [14, 15].

In response to the TG13, the World Society of Emergency Surgery (WSES) released comprehensive guidelines in 2016 with more specific recommendations regarding antibiotic choice, identification of high surgical risk patients, and alternative treatment options [1]. Similar to the TG13, it was recommended that, for those patients who are not surgical candidates, PTGBD should be performed despite acknowledging that high-quality prospective evidence for the procedure is lacking. While setting out to further define what factors may aid the surgical risk stratification, the WSES guidelines concluded that, due to a scarcity of evidence, no specific surgical risk stratification guidelines could yet be put forward.

Many studies have described PTGBD as a temporary measure to relieve gallbladder obstruction; however, there are conflicting results regarding the efficacy and complication rates of the procedure [14–17]. To date, there has been no randomized controlled trial comparing PTGBD to laparoscopic cholecystectomy or alternative nonsurgical treatment options except for the ongoing but incomplete CHOCOLATE trial [3] comparing laparoscopic cholecystectomy to PTGBD in an attempt to fill this gap in the literature.

Other nonsurgical treatment options are mentioned in the TG13, none of which have high quality evidence, and all of which have limitations. Gallbladder needle aspiration under ultrasound guidance is an alternate temporary measure that usually requires repeated punctures to be effective [1, 18] and has been shown to be clinically inferior to PTGBD [19]. Endoscopic nasogallbladder drainage and stenting is another well-described option in the literature but has been shown to be a difficult technique with suboptimal success rates [13, 20–25]. Endoscopic gallbladder drainage and/or stenting via the antrum of the stomach under ultrasound guidance has been performed [13]. It may have a role when biliary tree anatomy makes it difficult to traverse the cystic and common bile ducts. One prospective [26] and 9 retrospective [27–35] analyses have shown that technical and clinical success rates are close to 100%. However, the incidence of complications across the 10 studies (including pneumoperitoneum, stent migration, and bile leakage with no cases of biliary peritonitis) was approximately 10%. The limitations of these studies were that they were mostly undertaken with small cohort numbers (1–30 patients, mean 7.5), they were mostly retrospective nonrandomized studies, and the patients were not followed up long-term.

To date, there has been little research around the placement of permanent metallic stents along the cystic and common bile ducts; however, the evidence that exists is promising. Sheiman et al. [36] published a case report in which a percutaneous metallic stent was placed across the cystic duct to successfully relieve malignant obstruction, and the patient was symptom-free at five months. Miyayama et al. [37] used metallic stent placement in three patients with cholecystitis secondary to malignant obstruction (two patients had cholangiocarcinoma, and one had pancreatic carcinoma). The cholecystitis and associated symptoms improved in all patients. Two patients were symptom-free until they passed away at 3 and 10 months, and the surviving patient was still free of gallbladder symptoms 22 months after the procedure.

Brown et al. [38] used metal stents that traversed the cystic and common bile ducts from the gallbladder to the duodenum in five patients to treat acute cholecystitis secondary to both benign and malignant causes. Symptoms resolved in all patients, and stents remained patent for as long as 22 months at follow-up, with no cases of stent occlusion, migration, or recurrent cholecystitis. One patient had recurrent symptoms at three months; however, a cholecystogram demonstrated appropriately positioned, patent, and fully deployed stents.

Despite the positive results of the case studies so far, cholecystoduodenal stent insertion can be a technically challenging procedure with potential complications. Guide wires may be prevented from passing through the cystic duct into the bile duct due to the spiral valves or the presence of a cystic duct stricture. When a guide wire cannot be positioned appropriately, the procedure may need to be aborted. The durability of bare metal cholecystoduodenal stents may also be compromised by stent obstruction or occlusion. Recurrent cholecystitis in the setting of a stent may necessitate PTGBD. The presence of a cholecystoduodenal stent may make future endoscopic access to the biliary tree difficult should the patient develop cholangitis or biliary obstruction. This may necessitate a transhepatic approach to reaccess and decompress the biliary tree or cholecystoduodenal stent. Stent migration and viscus perforation have also been reported as rare complications from the use of metallic stents.

In our case, we performed cholecystoduodenal stenting to enable durable, antegrade gallbladder drainage into the duodenum. A theoretical concern with cystic duct stenting only as opposed to our approach of a stent from the cystic duct traversing the ampulla is the risk of reobstruction or occlusion. Deployment of the distal end of the cystic duct stent in the bile may cause funneling of the stent and obstruction. This is avoided by having the distal end of the stent in the duodenum as the stent is able to fully open and deploy adequately and widely in the duodenum.

Cholecystoduodenal stenting is a minimally invasive, potentially durable approach to managing recurrent cholecystitis in the elderly, comorbid patient. A small number of case studies thus far have demonstrated low complication rates. It is a justifiable approach in elderly, nonoperative patients in whom recurrent cholecystitis poses an ongoing problem, and where a permanent cholecystostomy is poorly tolerated. It requires expert interventional radiology input and is appropriate in highly select patients after careful consideration of their risk-benefit profile.
